# Supplementary material for: Identifying triplet pathways in dilute pentacene films
Source: Nat Commun. 2018 Oct 11;9:4222. doi: 10.1038/s41467-018-06330-x (PMC6181988; doi:10.1038/s41467-018-06330-x)
Supplement: Supplementary file 1 — Supplementary information [file 41467_2018_6330_MOESM1_ESM.pdf]

Supplementary Information

"Identifying Triplet Pathways in Dilute Pentacene Films"

D. Lubert-Perquel *et al.*

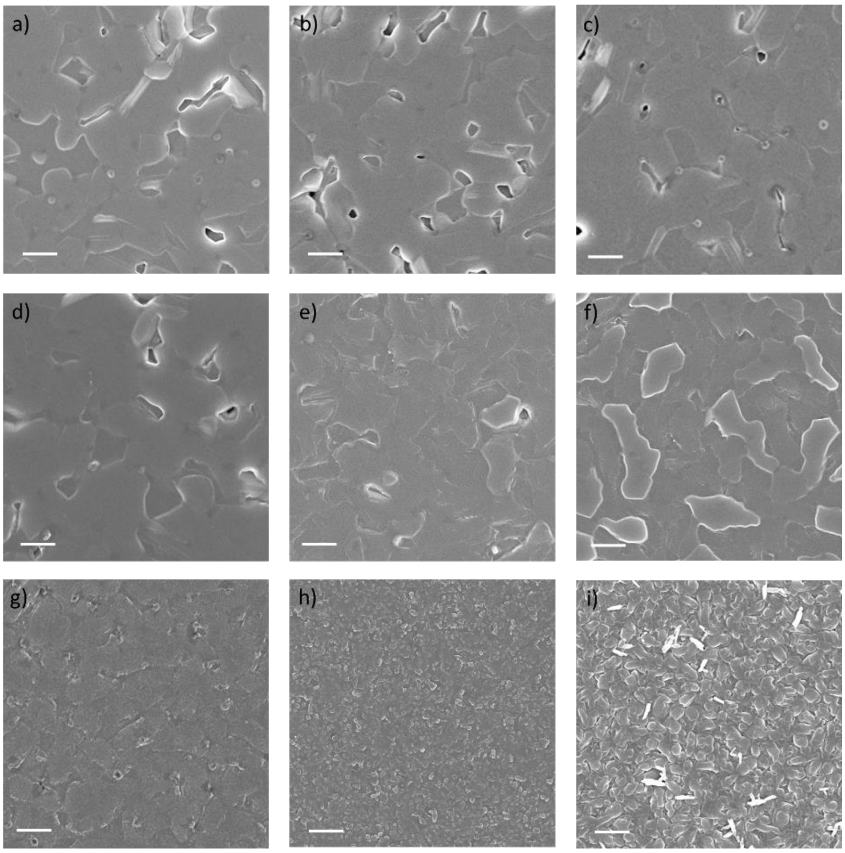

Supplementary Figure 1. a -j, show top view SEM images with a 1um scale bar. In order these are 0.1%, 0.2%, 0.5%, 1%, 2%, 5%, 10%, 20% and 50% pentacene dopant concentration in the p-terphenyl host. In the dilute concentrations platelets are observed like in pure *p*-terphenyl, however from 2% pentacene, fragmentation starts to occur and at 20% there is a clear reduction in grain size.

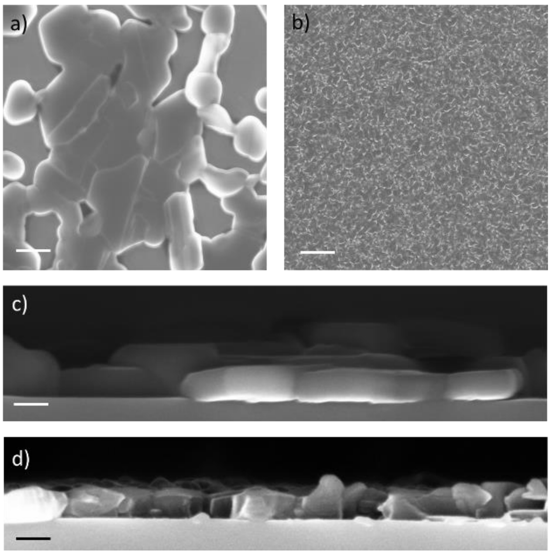

Supplementary Figure 2. **a,** Top view of p-terphenyl and **b,** pentacene with a 1μm scalebar. **c,** and **d,** are the corresponding cross-sections with a 200nm scale bar. These films have a nominal thickness of 200 nm. *p*-Terphenyl forms micron sized platelets whereas pentacene grows in grains an order of magnitude smaller.

Supplementary Note 1.

Optical Spectroscopy

Davydov splitting as a function of concentration was extracted to compare with previous studies. Broch *et al.* diluted pentacene in a matrix with lattice parameters larger than the pure pentacene structure[1], in contrast to the *p*-terphenyl used here, which has a smaller unit cell. Despite a reduction in lattice parameters the same trend as Broch *et al.* is observed, that is an increase in concentration yields an increase in Davydov splitting. In addition, the position of the $S_0 \rightarrow S_1$ energies is also reported to emphasize the lack of intermolecular electronic coupling below 1%, which gradually increases with the higher pentacene content as discussed in the main text.

The spectral changes outlined in Supplementary Fig. 3 indicate that intermolecular interaction strength increases with pentacene concentration, in addition to the higher proportion of pentacene aggregates intuitively expected from statistical mixing (note that below 10% pentacene any aggregated species lie below XRD sensitivity). At first glance, this conclusion appears to contradict the corresponding increase in lattice spacing (smaller diffraction angles, see main text Fig. 2c). However, given that XRD was performed on thin films in the 2θ configuration, and that both pentacene and *p*-terphenyl are orientated approximately perpendicular to the substrate, diffraction peaks correspond to long axis rather than π-π stacking.[2] We suggest that the latter distance decreases with higher pentacene concentrations due to their greater planarity relative to *p*-terphenyl, thus enhancing π-π interactions and hence splitting magnitude.

Fluorescence is quenched with increasing concentration of pentacene due to singlet fission outcompeting the radiative decay. The 0.1% spectrum does not follow the trend simply as there are too few pentacene molecules excited to emit a strong fluorescence.

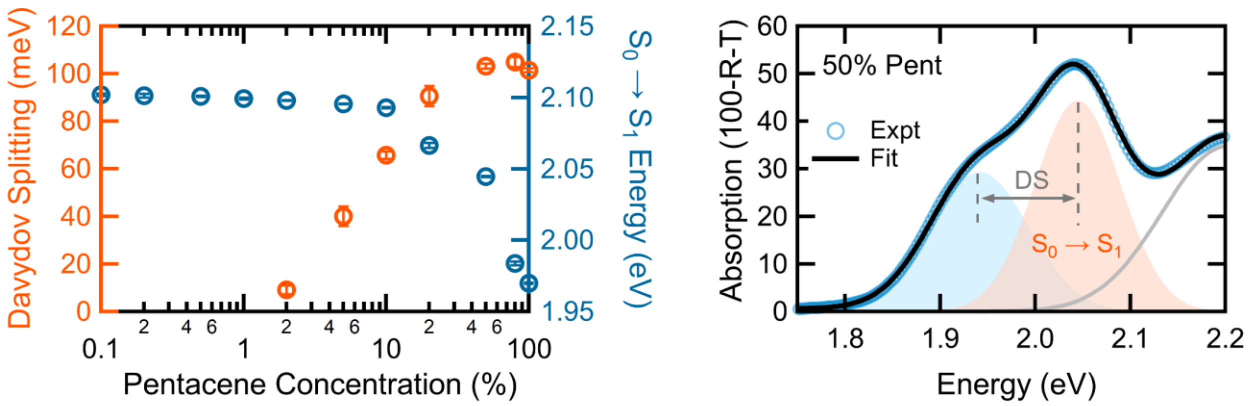

Supplementary Figure 3. Davydov splitting of the lowest energy absorption peaks with $S_0 \rightarrow S_1$ energies shown as a function of pentacene concentration. The 50% concentration fit is also shown as an example. Note that Davydov splitting becomes negligible at 2% pentacene and below, attributed to the lack of proximate pentacene molecules.

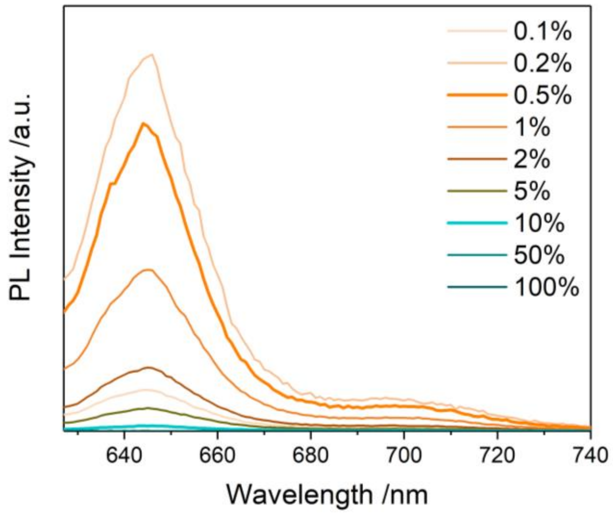

Supplementary Figure 4. Fluorescence spectra of the pentacene doped p-terphenyl films as a function of concentration, following excitation at 590 nm

Supplementary Note 2.

Molecular Orientation

An excited triplet state of an aromatic molecule, such as pentacene, is characterised by the presence of two unpaired electrons. There exists a dipolar interaction between such electrons that lifts the degeneracy of the three triplet sub-levels even in the absence of an applied magnetic field. Hence this interaction is named Zero Field Splitting (ZFS). The ZFS tensor is second rank and traceless, thus just two independent parameters, *D* and *E*, suffice to describe its components. The external magnetic field applied during an EPR experiment causes the additional Zeeman splitting of the triplet sub-levels. The usual EPR selection rule $\Delta m_S = \pm 1$ applies and therefore there exist two allowed EPR transitions (i.e. $m_{S(-1)}$ -> $m_{S(0)}$ and $m_{S(0)}$ -> $m_{S(+1)}$) for each molecular orientation, x, y and z as described in the main text. If the applied field is parallel to one of the ZFS axis, condition easily achieved with oriented samples, the splitting between the EPR lines allows determination of the ZFS parameter. Alternatively, if the ZFS parameters are known as it is the case for pentacene, the molecular orientation with respect to the applied magnetic field can be inferred by the line splitting. It is this latter approach that has been used in the present work. Supplementary Fig. 5a reports the energy level diagrams for pentacene triplet with the applied magnetic field parallel to the x-axis. In this case the energy of the x-axis is unchanged whereas the y- and z- axis mix and split proportionally to the applied field and the separation between the two EPR transitions corresponds to $|D|+3|E|$. Similarly, Supplementary Fig. 5b reports the energy level diagrams for the y- and z-axes parallel to the applied magnetic field, with transitions appearing at $|D|-3|E|$ and $2|D|$ respectively. From the above it follows that the two inequivalent sites in the *p*-terphenyl will have distinct angular dependences. This is demonstrated for isolated pentacene by the rotation pattern of the 0.5% film reported in Figure 4a (main text), which was used to establish the orientation and the degree of order of pentacene with respect to the substrate. When dimers and higher order aggregates with slightly modified ZFS parameters are present, as it is the case for pentacene concentrations larger than 1%, parallel and herringbone configurations can again be assigned on the basis of the measured angular dependency.

Dimer Structures

Two conformations of dimers (Supplementary Fig. 6) – herringbone with an average separation of 3.9 Å and parallel, either head-to-tail or side by side, both with a separation of 3.9 Å and 3.7 Å respectively. The values are an estimate based on the pentacene crystal structure[3] and some variations within a mixed structure are expected.

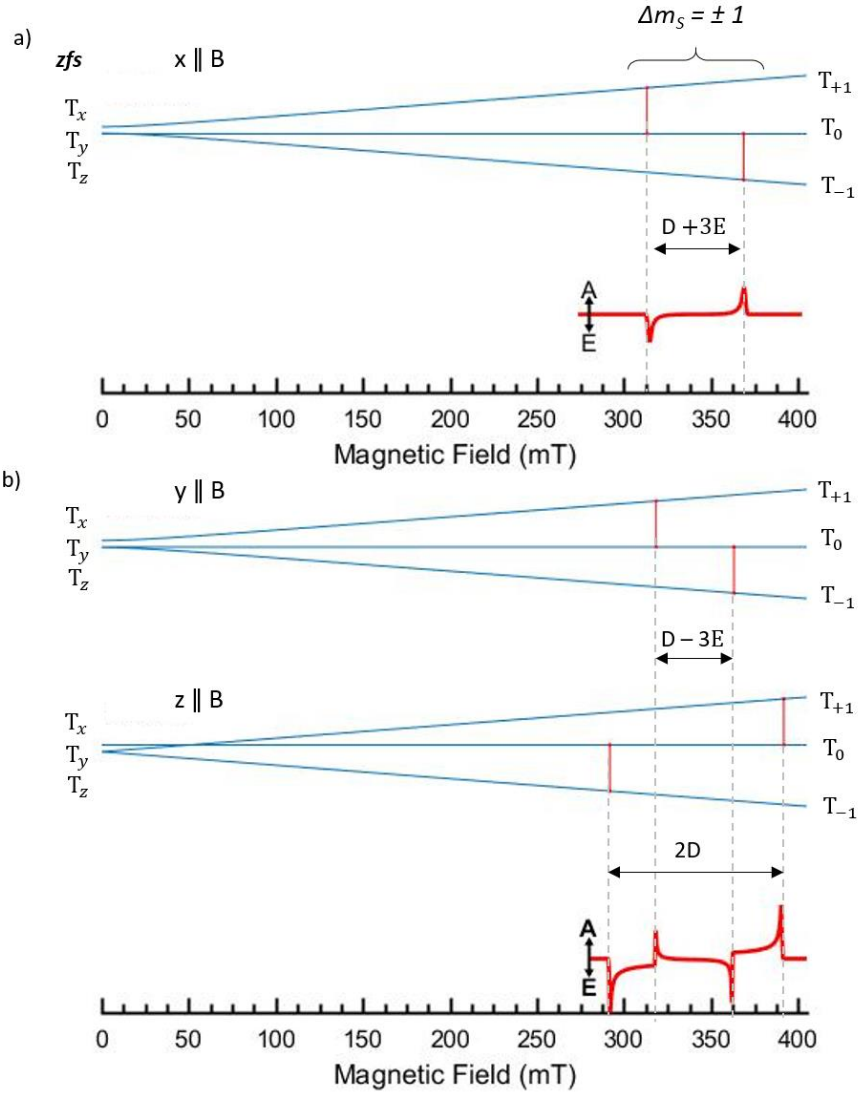

Supplementary Figure 5. Zeeman splitting of the pentacene molecule with **a,** x or **b,** y and z parallel to the applied magnetic field. Simulations are shown with the peaks appearing at the corresponding resonance field positions.

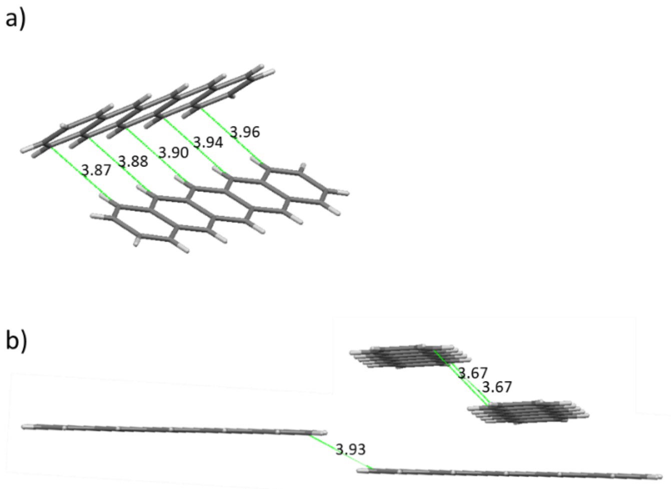

Supplementary Figure 6. a, The herringbone and **b,** parallel dimer conformations. The herringbone dimer has an average separation of 3.9 Å and the parallel dimer 3.8 Å as determined from .cif file reference PENCEN10[3].

Supplementary Note 3.

Transient Nutation Experiment

Transient nutation is a pulsed EPR experiment that is used to disentangle overlapping spectra; it exploits the dependence of the nutation frequencies on the transition matrix elements, determined by the total spin S and the spin projection component m_s, and the strength of the applied microwave field. As a result, if the microwave field is kept constant, nutation frequencies allow for a definitive assignment of the different transitions based on spin quantum numbers[4,5]. The explicit formula correlating the nutation frequency to the total spin and the spin projections characteristic of the transition reads:

$$\omega_{m_S,m_{S\pm1}} = \omega_{1/2}\sqrt{S(S+1) - m_S(m_S \pm 1)} \quad (1)$$

where $\omega_{1/2} = g\mu_B B_1/\hbar$ is the precession frequency for a spin S = 1/2 system and B_1 is the magnetic component of the microwave field in the resonator. The ratio of the triplet (subscript T) to quintet (subscript Q) nutation frequency is given by:

$$\omega_{Q/T} = \sqrt{\frac{S_Q(S_Q+1) - m_{S_Q}(m_{S_Q}\pm1)}{S_T(S_T+1) - m_{S_T}(m_{S_T}\pm1)}} \quad (2)$$

Each spin system will have its own nutation frequency. It follows that for transitions from the $m_s = 0$ state, the ratio of nutation frequencies for pure quintet and triplet states is $\sqrt{3}$. Conversely, for transitions from the $m_S = 1$ the ratio equates to $\sqrt{2}$. Comparison between measurements at different field positions but with all the other experimental settings unchanged (note that nutation frequencies depend on the strength of B_1 and therefore on the Q factor) provides a way of probing the spin state of the observed transitions.

Supplementary Note 4.

The Spin Hamiltonian

The Hamiltonian for the system is given as the sum of the Zeeman, dipolar and exchange interactions as follows:

$$\hat{\mathcal{H}} = \mu_B g \sum_i \sum_j B_j \hat{S}_{ij} + h\left[\sum_i D\left(\hat{S}_{iz}^2 - \frac{1}{3}\hat{S}_i^2\right) + E\left(\hat{S}_{ix}^2 - \hat{S}_{iy}^2\right) + \sum_j \sum_k J_{jk}\, \hat{S}_{1j}\hat{S}_{2k}\right] \quad (2)$$

where the μ_B is the Bohr magneton, h is Planck's constant, g is the Landé g-factor and J is the exchange coupling. D and E are the zero-field splitting parameters and the spin operators of each triplet, $\hat{S}_i$, where the index $i = 1, 2$, j and k denote the Cartesian coordinate system x, y, z. As has been previously reported the J tensor is the sum of its isotropic and anisotropic components, which in turn is composed of a symmetric and antisymmetric component[4,6]. For the herringbone dimer configuration, a purely isotropic J was fitted, whereas the parallel dimer had a large anisotropic contribution, as detailed below. The antisymmetric component of the anisotropic J was always set to 0 and the symmetric component was described by the traceless matrix[4,6]

$$J_{aniso} = \begin{pmatrix} X & 0 & 0 \\ 0 & X & \alpha X \\ 0 & \alpha X & -X(1+A) \end{pmatrix} \quad (3)$$

where α, A and X are fine structure parameters defined as $A = 1 - 3\,sin^2\,\varphi$; $\alpha = \sqrt{(1-A)(2+A)}$ and φ is the angle between the z-axes of the zfs tensor of the chromophores, which was set to 0 for the collinear dimers. This therefore reduces to the diagonalized $J_{aniso} = [1\;1-2]X$.

Tr-Epr Fitting of the Pentacene Doped *P*-Terphenyl

The software package Easyspin[7] was used to simulate strongly coupled triplets in the three distinct configurations: monomer, herringbone dimer and parallel dimer. The zero-field splitting parameters of the monomer and parallel dimers were fit to the previously reported pentacene values[8], defined as shown in Supplementary Fig. 5. However, the herringbone dimer showed increased splitting. This is discussed in the main text and is likely due to the angle between molecules leading to the variations in the dipolar coupling. As previously mentioned, the herringbone dimer contribution was fitted with a purely isotropic J-coupling. However, for the parallel dimer contribution this was insufficient to effectively represent the system and so an anisotropic component was included. The initial parameters were set similarly to dimers linked in the x-axis of the zero-field tensor[4]. However, the fitted parameter showed a larger anisotropic component, which could be attributed to the morphology of the sample, though further investigation is required.

The parameters are detailed in Supplementary Table 1, with the isotropic J-values as thresholds rather than precise calculations.

Supplementary Table 1. Fitting parameters of the strongly coupled triplets.

		D	E	J_{iso}	J_{aniso}	lifetime
Herringbone Dimer	Quintet	1600	80	20GHz	-	~500ns
Parallel Dimer	Quintet	1400	50	20GHz	60MHz	~300ns
Single Molecules	Triplet	1400	50	N/A	N/A	>1us

Tr-Epr Fitted Spectra of the y,z-Orientation

The left panel of Supplementary Fig. 9 shows the y,z-orientation (110°) as a function of pentacene concentration. The signal is largest for the 0.5%, as expected from the x-orientation, which also shows the strongest preferential orientation. An additional contribution can be noticed at 1% indicative of increased texture, likely corresponding to a different orientation in the system. The disorder is increased with increasing concentration until the polycrystalline quintet spectrum is resolved at 10%. The quintet contributions are shown in the shaded grey area, with increasing concentration additional peaks are observed due to disordered contributions. The signal was too weak above 10% to resolve the last two concentrations. The innermost pair of peaks were identified as an x-orientation component which is not present in very dilute samples.

The right panel of Supplementary Fig. 9 simulates the 10% pentacene doped *p*-terphenyl in the y,z-orientation parallel to the applied field. An S = 2 model with a large ordering parameter is used and all contributions are resolved this way.

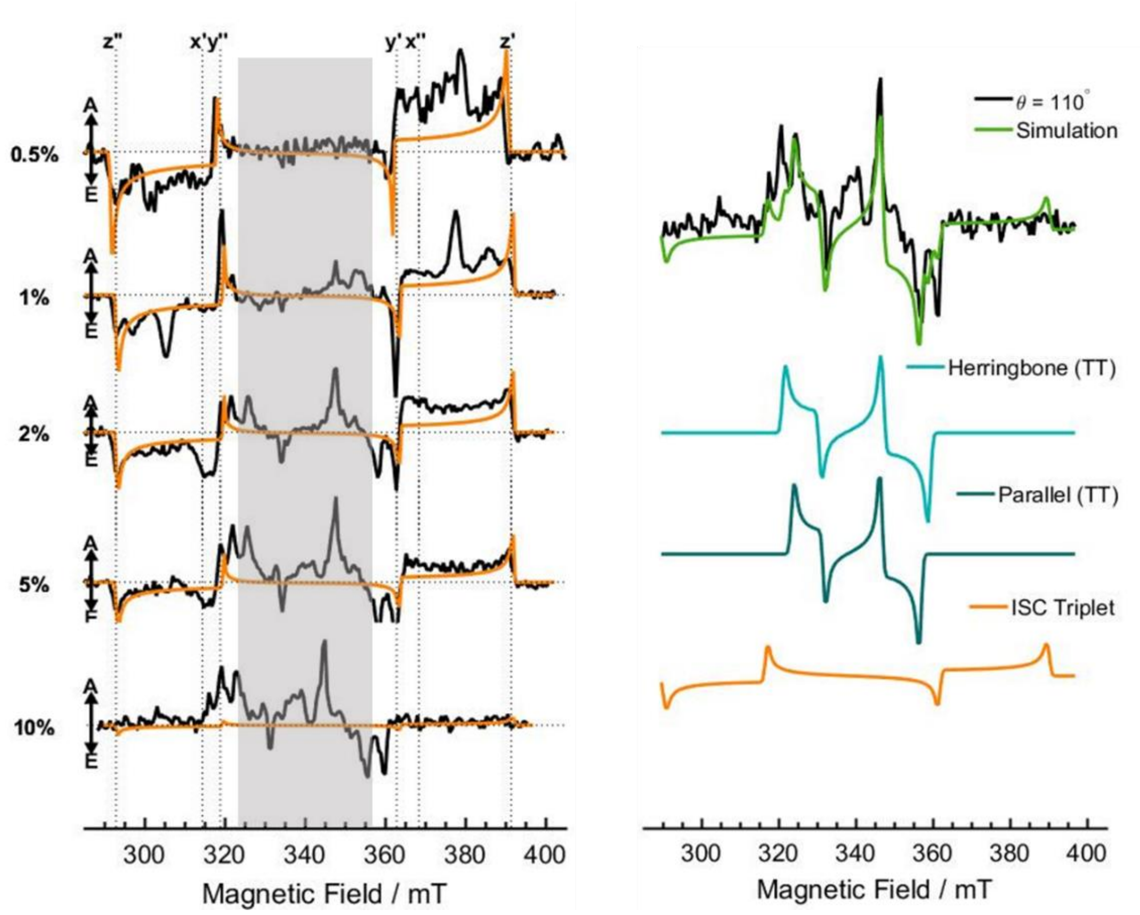

Supplementary Figure 7. (Left) Time-resolved EPR spectroscopy at 110° between the normal to the substrate and the magnetic field as a function of concentration. (Right) TR-EPR of the 10% pentacene doped p-terphenyl film with S=2 simulations for the quintets and the ISC triplet contribution. Spectral slices were taken at the maximum signal, time 500 ns.

<u>**Supplementary Note 5.**</u>

Kinetic Model of the Time Evolution of Tr-Epr Time Traces

A kinetic model was drawn up to determine the lifetimes of the spin states. The parallel and herringbone configurations were treated separately, though both were solved using the same set of coupled differential equations.[9] A schematic of the kinetic model is shown in Supplementary Fig. 10, with corresponding spin-state populations shown below. The sole difference between the two orientation is that an ISC triplet contribution has to be included at the resonance positions of the parallel configuration. The instrument response, k_{IRF}, was estimated at 200ns and kept constant throughout the analysis. To determine the time response of the system, 4 rates were defined: i) the dissociation of the quintets into free triplets, ^{5}(TT) → T + T, is defined with the rate constant k_{diss}; ii) the back reaction, T + T → ^{5}(TT), with a rate of constant of k_{TQ}; iii) the decay of the quintet to the ground state due to geminate back recombination and defined with a rate constant k_{rec}; and iv) a phenomenological rate constant for the decay of the triplet to the ground state, k_{IC}, which include all the relaxation processes (spin-lattice relaxation and recombination) that cannot be distinguished. We note that spin-lattice relaxation is unlikely to be the dominating contribution since the measured tr-EPR spectra decay to zero before reaching Boltzmann. The modelled time traces are shown in the main text Figure 7c and d and a summary of the rates derived from the model is reported in Supplementary Table 2.

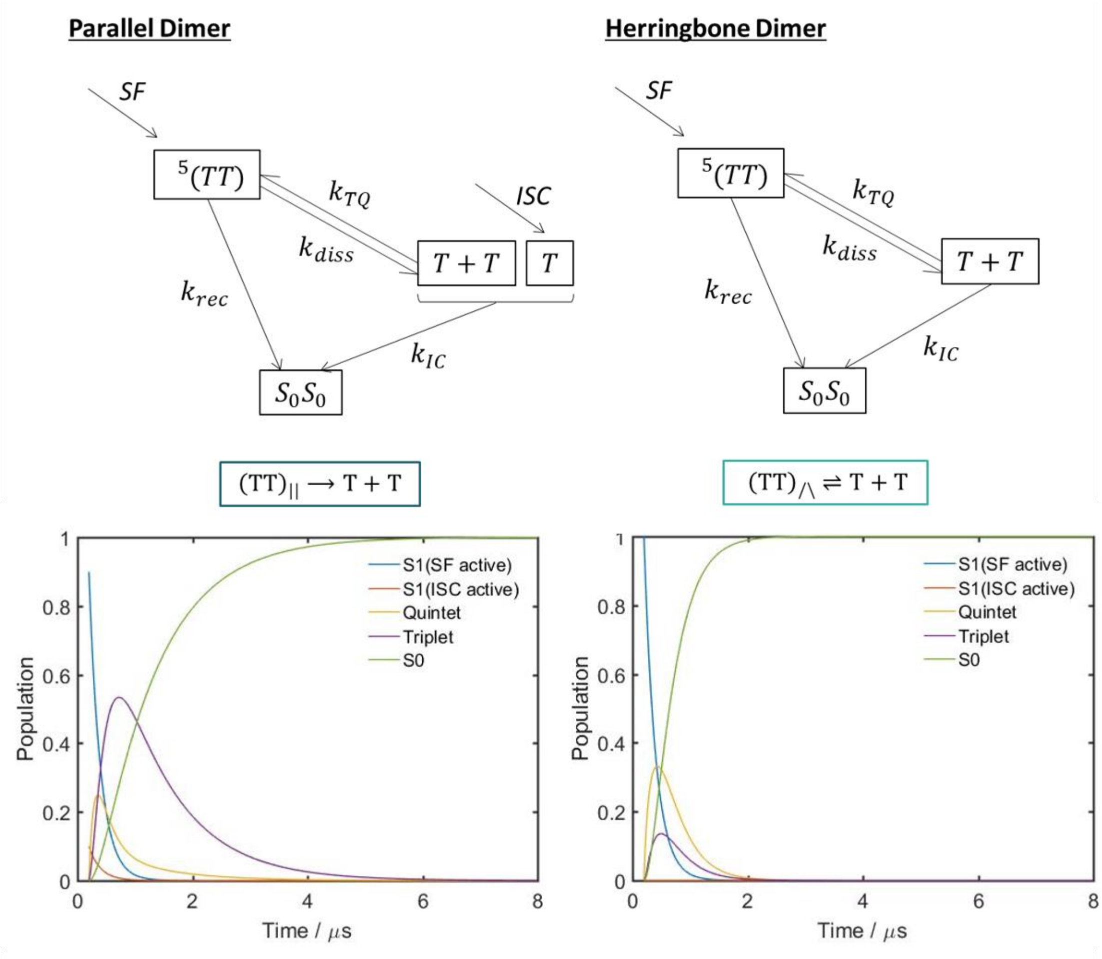

Supplementary Figure 8. Schematic of the quintets and triplets with rate constants, according to their geometries, with corresponding spin populations.

Supplementary Table 2. Fitting parameters of the four decays of the kinetic model for each dimer geometry.

	Parallel Dimer Rates (s^{-1})	Herringbone Dimer Rates (s^{-1})
k_{diss}	8.0×10^6	8.0×10^6
K_{rec}	1.0×10^6	3.0×10^6
K_{TQ}	8.0×10^5	1.6×10^7
K_{IC}	1.0×10^6	3.0×10^6

Supplementary References

1. Broch, K. *et al.* Robust singlet fission in pentacene thin films with tuned charge transfer interactions. *Nat. Commun.* **9,** 954 (2018).

2. Morisaki, H. *et al.* Large surface relaxation in the organic semiconductor tetracene. *Nat. Commun.* **5,** 1–6 (2014).

3. Siegrist, T. *et al.* A polymorph lost and found: The high-temperature crystal structure of pentacene. *Adv. Mater.* **19,** 2079–2082 (2007).

4. Tayebjee, M. J. Y. *et al.* Quintet multiexciton dynamics in singlet fission. *Nat. Phys.* **13,** 182–188 (2016).

5. Weiss, L. R. *et al.* Strongly exchange-coupled triplet pairs in an organic semiconductor. *Nat. Phys.* **1,** (2016).

6. Benk, H. & Sixl, H. Theory of two coupled triplet states application to bicarbene structures. *Mol. Phys.* **42,** 779–801 (1981).

7. Stoll, S. & Schweiger, A. EasySpin , a comprehensive software package for spectral simulation and analysis in EPR. *J. Magn. Reson.* **178,** 42–55 (2006).

8. Yang, T. C., Sloop, D. J., Weissman, S. I. & Lin, T. S. Zero-field magnetic resonance of the photo-excited triplet state of pentacene at room temperature. *J. Chem. Phys.* **113,** 11194–11201 (2000).

9. Sakai, H. *et al.* Multiexciton Dynamics Depending on Intramolecular Orientations in Pentacene Dimers : Recombination and Dissociation of Correlated Triplet Pairs. *J. Phys. Chem. Lett.* **9,** 3354 (2018).
